# Elexacaftor–Tezacaftor–Ivacaftor Therapy for Cystic Fibrosis Patients with The F508del/Unknown Genotype

**DOI:** 10.3390/antibiotics10070828

**Published:** 2021-07-07

**Authors:** Marika Comegna, Vito Terlizzi, Donatello Salvatore, Carmela Colangelo, Antonella Miriam Di Lullo, Immacolata Zollo, Giovanni Taccetti, Giuseppe Castaldo, Felice Amato

**Affiliations:** 1 Department of Molecular Medicine and Medical Biotechnologies, University of Naples Federico II, Via Pansini, 5, 80131 Naples, Italy; marika.comegna@unina.it (M.C.); immacolata.zollo@unina.it (I.Z.); giuseppe.castaldo@unina.it (G.C.); 2 CEINGE–Advanced Biotechnologies, Via G. Salvatore, 486, 80145 Naples, Italy; 3 Cystic Fibrosis Regional Reference Center, Department of Pediatric Medicine, Anna Meyer Children’s University, Viale Pieraccini, 24, 50139 Florence, Italy; vito.terlizzi@meyer.it (V.T.); g.taccetti@meyer.it (G.T.); 4 Cystic Fibrosis Center, Hospital San Carlo, Via P. Petrone, 85100 Potenza, Italy; saverdon@gmail.com (D.S.); c.colangelo@tiscali.it (C.C.); 5 Department Reproductive Sciences and Dentistry, University of Naples Federico II of Neuroscience, Via Pansini, 5, 80131 Naples, Italy; antonellamiriam.dilullo@unina.it

**Keywords:** theratyping, cystic fibrosis, functional characterization, personalized medicine, CFTR, rare mutation

## Abstract

The new CFTR modulator combination, elexacaftor/tezacaftor/ivacaftor (Trikafta) was approved by the FDA in October 2019 for treatment of Cystic Fibrosis in patients 6 years of age or older who have at least one F508del mutation in one allele and a minimal-function or another F508del mutation in the other allele. However, there is a group of patients, in addition to those with rare mutations, in which despite the presence of a F508del in one allele, it was not possible to identify any mutation in the other allele. To date, these patients are excluded from treatment with Trikafta in Italy, where the CF patients carrying F508del/unknown represent about 1.3% (71 patients) of the overall Italian CF patients. In this paper we show that the Trikafta treatment of nasal epithelial cells, derived from F508del/Unknown patients, results in a significant rescue of CFTR activity. Based on our findings, we think that the F508del/Unknown patients considered in this study could obtain clinical benefits from Trikafta treatment, and we strongly suggest their eligibility for this type of treatment. This study, adding further evidence in the literature, once again confirms the validity of functional studies on nasal cells in the cystic fibrosis theratyping and personalized medicine.

## 1. Introduction

Cystic fibrosis (CF) is an autosomal recessive genetic disease characterized by mutations in the cystic fibrosis transmembrane conductance regulator (CFTR) gene [1]. To date, 360 CFTR mutations are known to be CF-causing (https://cftr2.org/, accessed on 31 July 2020), that are classified according to the impact they have on the synthesis, processing, or function of the CFTR gene [2]. New drugs that target the basic defect in CF have provided hope for patients and progress in drug development has been substantial over the past decade. The first class of molecular drugs successfully developed were CFTR potentiators, such as ivacaftor, which are small molecules that interact with the mutant channel to augment its opening probability, enhancing anion flux across the plasma membrane [3,4]. At present, ivacaftor (Kalydeco^TM^) is approved in the US for patients aged 4 months and older, carrying gating mutations and mutations with residual CFTR function [3,5]. CFTR correctors (e.g., elexacaftor, lumacaftor, tezacaftor) correct the processing and trafficking defect of the F508del-CFTR protein to enable it to reach the cell surface. The combination of a single corrector, either lumacaftor or tezacaftor, with the potentiator ivacaftor improves clinical outcomes, including lung function and the rate of pulmonary exacerbations [6,7]. Actually, the Food and Drug Administration (FDA) has approved the use of lumacaftor/ivacaftor (Orkambi^®^) in patients with CF who have two copies of the F508del CFTR mutation and are 2 years and older; in the same way tezacaftor/ivacaftor (Symdeko^®^) is approved for individuals 6 years and older with two copies of F508del, as well as for individuals who have a single copy of one of 154 specified mutations, regardless of their other mutation [8]. The newest CFTR modulator elexacaftor/tezacaftor/ivacaftor (Trikafta) was approved by the FDA in October 2019 for the treatment of CF in patients 12 years of age or older who have at least one F508del mutation, including both heterozygous for the F508delCFTR mutation and a minimal-function mutation, and homozygous for the F508del mutation.

To date, In the US the combination of elexacaftor/tezacaftor/ivacaftor (TRIKAFTA^TM^) is a prescription medicine used for the treatment of cystic fibrosis (CF) in patients aged 6 years and older who have at least one copy of the F508del mutation in the CFTR gene or another of 177 mutations that are responsive in vitro to treatment with TRIKAFTA. In EU the combination elexacaftor/tezacaftor/ivacaftor (Kaftrio^TM^) is used in patients aged 12 years and older whose CF is due to at least one F508del mutation in the CFTR gene.

Thus, on the contrary of US and EU countries, where elexacaftor/tezacaftor/ivacaftor combination is approved for CF patients with at least one copy of F508del, regardless of the second identified (or unidentified) variant, in Italy the Kaftrio^TM^ therapy is not approved.

Approval was resulting from improvements in lung function, the rate of pulmonary exacerbations, sweat chloride concentration, CFQ-R respiratory domain scores, and BMI over 6-month testing periods in a phase-3 trial [9]. This triple combination helps the CFTR protein perform better than other modulators for an even greater number of patients with CF. Nevertheless, further research to extend the benefit of CFTR modulation to patients with responsive mutations other than the F508del CFTR mutation or rare genetic profiles is imperative.

The ability of the triple combination to rescue the mutated CFTR by the F508del mutation opens new perspectives for CF patients whose genotype is characterized by the presence of the F508del and another unknown mutation, remaining unidentified also after deep gene sequencing analysis. This F508del/unknown subgroup, usually not included in clinical trials, is represented by 71 patients in Italy (about 1.3% of the overall Italian CF patients, personal communication of Italian CF Registry) [10], and some of them suffer of severe lung disease.

Aim of our paper was to evaluate the efficacy of the triple therapy Kaftrio^TM^ on nasal epithelial cells (hNECs) of three CF adult patients carrying a CFTR genetic profile F508del/unknown.

## 2. Results

### 2.1. Patients Characterization

We report three cases of CF unrelated patients with F508del/unknown genotype. For all the three subjects, the CFTR gene analysis was performed at diagnosis.

Case 1 a 48-year-old Caucasian woman diagnosed as CF with pancreatic insufficiency (fecal elastase <100 µg/g) at the age of 8 years old in presence of persistent productive cough, diarrhea and pathological sweat chloride (chloride values: 104–102 mEq/L). During follow up she developed chronic colonization by Pseudomonas Aeruginosa, and by Burkholderia gladioli. Further complications were CF-related diabetes mellitus and non-cirrhotic CF-related liver disease. The clinical course of the lung disease worsened progressively, needing with nocturnal oxygen therapy since the age of 36 years. Forced expiratory volume in 1 s (FEV1) values dropped <30% in the last year. Finally, the patient suffered one episode of major hemoptysis (acute bleeding of more than 240 mL/day) requiring bronchial artery embolization at the age of 37 years.

Case 2 a 40-year old Caucasian man, diagnosed as CF with pancreatic sufficiency at the age of 9 years, on the basis of chronic productive cough, evidence of bronchiectasis at CT-scan and colonization by Pseudomonas aeruginosa. Sweat chloride at diagnosis was pathological (82 mEq/L). When adolescent, the clinical picture was reinforced by the evidence of azoospermia. The clinical course of the lung disease was progressively worsening, with acquisition of multi-resistant *P. aeruginosa* and very frequent pulmonary exacerbations. The subject initiated long-term therapy with oxygen at the age of 38 years, and at the age of 39 entered on the waiting list for lung transplantation, because of recurrent massive haemoptysis, not responding to bronchial artery embolization.

Case 3 a 59-year old Caucasian woman, diagnosed as CF with pancreatic sufficiency at the age of 20 years old, on the basis of chronic productive cough, evidence of bronchiectasis at CT-scan and colonization by *P. aeruginosa*. Sweat chloride at diagnosis was pathological (114 mEq/L). The clinical course of the lung disease was progressively worsening, with acquisition of multi-resistant *P. aeruginosa* and very frequent pulmonary exacerbations. The subject initiated long-term therapy with oxygen at the age of 57 years, and her lung function has declined to 28% in the last year.

All major clinical features are described in Table 1.

### 2.2. Nasal Brushing and Short-Circuit Current Recordings

**Figure 1:**
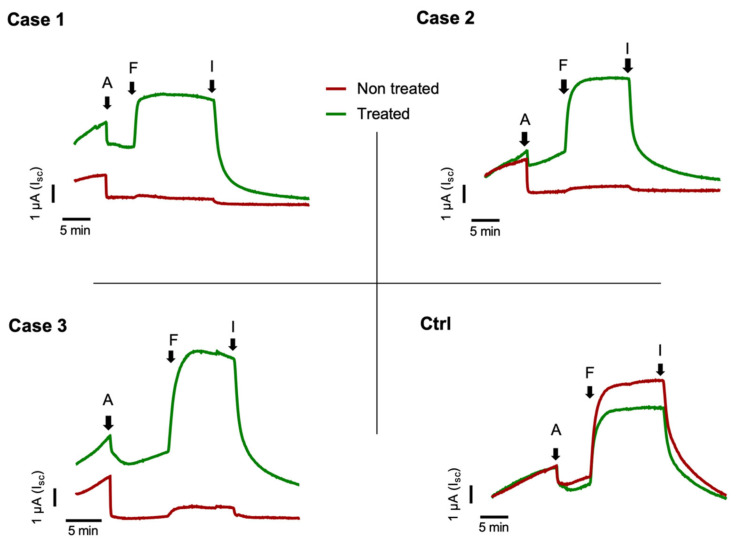
Short-circuit current (Isc) recording experiments on brushed nasal epithelial cells. Case 1 to 3 are short-circuit currents from CF patients with F508del/Unknown genotype treated with Kaftrio^TM^. Cells were treated with (green line) or without (dark red line) a combination of VX-445 (3 μM), VX-661 (3 μM) and VX-770 (100 nM) for 24 h. The black arrows indicate: *A* = addition of Amiloride (100 μM) for ENaC channel activity inhibition, *F* = addition of Forskolin (10 μM), IBMX (3-Isobutyl-1-methylxanthine) (100 μM) for activation of the transepithelial cAMP-dependent current (including CFTR channel activity) and *I* = addition of Inh-172 (5 μM) for CFTR specific inhibition. All chemicals were added to the apical/mucosal side of cells.

In order to evaluate the eligibility of our patients for the treatment with Kaftrio^TM^ therapy, we performed a nasal brushing to each of them and analyzed it by short-circuit current recordings. As shown in Figure 1 the untreated cells of F508del/Unknown patients did not respond to FSK-IBMX activation. On the contrary, the treatment of the cells with Kaftrio^TM^ results in an increase of current elicited by FSK-IBMX from 3 to 5 μA (Figure 1), similarly to non-CF subjects (Ctrl subject Figure 1). Addition of the CFTRinh-172 (an CFTR specific inhibitor) essentially abolished the current elicited by cAMP agonist. Figure 1 demonstrates, despite the clear effect of Kaftrio^TM^ treatment on the activity of the CFTR protein in these patients, that there is a heterogeneity in the Isc traces. This is probably due to the different genetic backgrounds of the various subjects, and therefore to the different CFTR protein interactome. In fact, it is now known that the activity of CFTR and the rescue of the F508del-CFTR protein activity may depend on the proteins with which it interacts [11,12]. Therefore, if on the one hand, the use of ex vivo models allow evaluating the drug responsiveness regardless of the patient’s genotype, on the other hand, the study of the CFTR interactome is of fundamental importance for the search for new therapeutic targets for cystic fibrosis.

**Figure 2:**
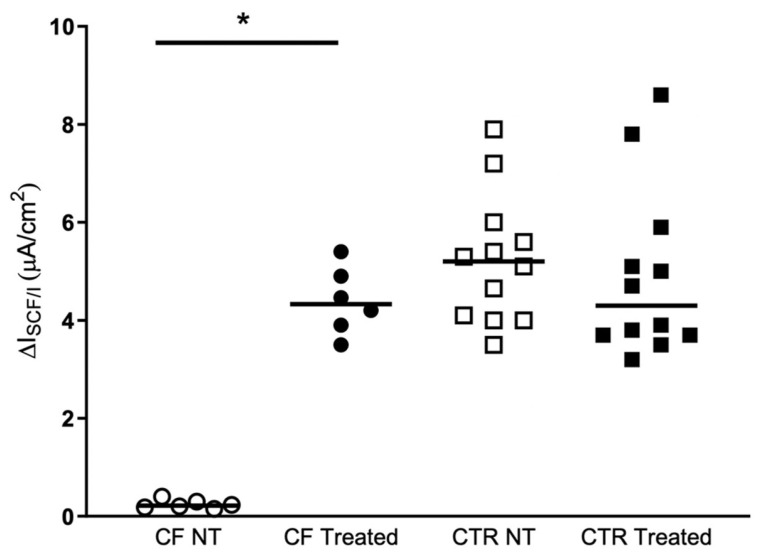
Kaftrio^TM^ effect on CFTR-dependent Cl-secretion. Isc response to FSK Forskolin (10 μM), IBMX (100 μM) (ΔIscF/I) after treatment for 24 h with DMSO (No-treated) or a combination of VX-445 (3 μM), VX-661 (3 μM) and VX-770 (100 nM) (Treated). ΔIscF/I recording experiments on brushed nasal epithelial cells from CF patients (two filters per patients, *n* = 3) with F508del/Unknown genotype and control subjects (two filters per subject, *n* = 6). CF NT: Cystic Fibrosis patients Non Treated (white circles), CF Treated (black circles), CTR NT: Control subjects Non Treated (white squares), CTR Treated (black squares). The *p*-values for statistically significant differences are indicated as * <0.031. In the Table 2 are presented the statistical analysis of the samples analyzed.

Figure 2 shows the current elicited by cAMP agonists in the hNECs derived from F508del/Unknown patients and from control subjects. There were no significant differences in treated cells compared to no treated ones in the control samples. On the contrary, when the hNECs with F508del/Unknown genotype were treated with Kaftrio^TM^ the current elicited by forskolin/IBMX was drastically increased respect to untreated cells, reaching values similar to control samples.

## 3. Discussion

The development of CFTR modulators is changing the natural history of cystic fibrosis. Approximately 90% of American people with CF carry one or more copies of the F508del-CFTR allele, thus making almost all patients eligible for new drugs [9]. Nevertheless, frequency and distribution of F508del varies in different population, with a decreasing prevalence from Northwest to Southeast Europe [13]. Data from Italian Cystic Fibrosis Registry show that only 21.1% of CF patients are homozygous for F508del CFTR mutation [6]. Furthermore only 45% of CF patients followed at CF center of Florence have at least one F508del [14]. Despite the wide availability of genome sequencing technologies, not in every CF patient can be identified a mutation on both CFTR alleles. This occurs in 1–1.5% CFTR alleles of patients with fully expressed disease from Northern Europe and in an even higher proportion of CFTR alleles from Southern Europe [7].

The Italian CF Registry has the records of 5501 CF patients in 2018. Out of the total, 3774 patients carry one allele with the F508del mutation. Within this last subgroup, 71 patients (about 1.3% of the Italian CF patients) have their genotype characterized by F508del mutation in one allele and unidentified mutation in the other allele. However, not for all the patients can be guaranteed that an extensive gene scanning has been performed. For these reasons, despite all efforts, the proportion of potentially eligible patients suitable for CFTR modulators varies between countries and there is still a subgroup of patients that are not rescued by any of the drugs in the development pipeline. In these specific cases novel drug screening needed. Currently international projects, including CF patients with rare mutations, are going to evaluate the efficacy of new molecules on nasal or intestinal organoid ex-vivo models.

In the setting of such studies, the concept of “minimal function” could be interpreted in an innovative way. We have studied several other patients with this approach [15,16] and recently it was possible to begin a treatment with ivacaftor in a subject with a rare mutation, on the basis of the demonstration of functional studies on nasal epithelial cells [17,18]. Here we show the use of epithelial nasal cells from patients as ex vivo model to evaluate the Kaftrio^TM^ responsiveness on three CF patients with F508del/unknown genotype. Our model of nasal epithelial cells is simple, standardizable and non-invasive for the patient [19,20]. In the present study, all three patients analyzed responded very well to treatment with Kaftrio^TM^. The second mutation was not identified also using deep sequencing; nevertheless, its effect should be quite severe, as untreated cells showed basically very low levels of CFTR activity (less than 3% of wild-type CFTR activity) after cAMP agonist stimulation.

Finally, with this work we stress the importance of this kind of ex vivo models for the functional characterization of CFTR rare mutations to predict the drugs responsiveness regardless of the CFTR genotype [18]. Based on our data, we add further evidence in the literature, confirming the validity of functional studies on nasal cells in the cystic fibrosis theratyping and personalized medicine.

Of course, our data do not suggest that all F508del/unknown patients are eligible for Kaftrio^TM^ treatment, as we do not know the exact genomic condition of our three patients and certainly each of the patients with this genotype has a different genomic condition. Full-gene CFTR sequencing could help to identify the second mutation possibly located in intronic or regulatory regions such as promoter [21] or 3’UTR [22] regions.

But since knowing the genetic cause of these patients requires complicated molecular analyzes, probably whole genome sequencing techniques, functional characterization using ex vivo models represent a simple and valid way to define their eligibility for treatment with available therapies. Indeed, ex vivo studies allow, unlike in vivo ones, where it is required to perform experiments on the whole organism, in this case, the patient, to predict the pharmacological responsiveness on few cells derived from the patient, and therefore, with the same genetic background, saving money, time and with great clinical benefit for patients.

## 4. Materials and Methods

### 4.1. Patients Characterization

For all the three subjects, the CFTR gene analysis was performed at diagnosis. The DNA samples were analyzed by three methods: (1) Inno-LiPA CFTR 27+TN kit Cat#: 80581, Inno-LiPA CFTR 19 kit Cat#: 80580, Inno-LiPA CFTR Italian regional kit Cat#: 80579, all kits are provided by FUJIREBIO (Göteborg, Sweden), total 56 mutation analyzed, (2) Sequencing analysis of all CFTR exons plus 100 bp up- and down-stream sequences, by Sanger method, (3) quantitative fluorescent multiplex PCR MLPA (Polymerase Chain Reaction Multiplex Ligation-dependent Probe Amplification) analysis for the detection of deletions, insertions and duplications in all coding regions. (detection rate 98%). All the tests showed exclusively the presence of the F508del mutation, and a not identified second mutation. Case1 was analyzed by NGS technology (Devyser CFTR, Stockholm, Sweden), Art. No.: 8-A101.

### 4.2. Nasal Brushing

Human nasal epithelial cells (HNEC) were sampled as reported in by nasal brushing of both nostrils. Briefly, brushes were immersed in RPMI 1640 medium supplemented with 3% Penicillin-Streptomycin for cell culture. Then, each sample were incubated for 20 minutes in a thermomixer and in agitation at 600 rpm to detach all cells from brushes and resuspended in PneumaCult –EX Medium (STEMCELLCat#: 05008), a serum free cell culture medium, and seeded in a T12.5 collagen-coated flasks. Cell culture medium was changed every day. Then, about 33,000 cells (1 × 105 cells/cm^2^) were seeded on porous filters (0.33 cm^2^, Transwell, Corning Cat#: 3470) in PneumaCult–EX Medium until confluence. The PneumaCultTM–EX Medium was replaced by PneumaCult-ALI Maintenance Medium (STEMCELL Cat#: 05022) for air-liquid interface (ALI) cultures.

### 4.3. Short-Circuit Current Recordings

After at least 20 days culture, or when the TEER value is ≥600 Ω·cm^2^ [23], the function of epithelial nasal tissue is tested by Ussing Chambers, as reported in Amato et al. HM 2019 [17] with some variations. Both apical and basolateral hemi-chambers were filled with 5 ml of a solution containing (in mM): 126 NaCl (Sodium Chloride), 0.38 KH_2_PO_4_ (Potassium dihydrogen phosphate), 2.13 K_2_HPO_4_ (Dipotassium hydrogen phosphate), 1 MgSO_4_ (Magnesium Sulphate), 1 CaCl_2_ (Calcium Chloride), 24 NaHCO_3_ (Sodium Bicarbonate), and 10 glucose, final pH 7–7.3. Both sides were continuously bubbled with a gas mixture containing 5% CO_2_ (Carbonic Anhydride) −95% air and the temperature of the solution was maintained at 37 °C. The transepithelial voltage was short-circuited with a voltage-clamp (VCC MC8 Multichannel Voltage/Current Clamp, Physiologic Instruments). The offset between voltage electrodes and the fluid resistance were canceled before experiments. The short-circuit current was acquired and analyzed using the Acquire & Analyze software Version 2.3.8 (Physiologic Instruments), the raw tracings were processed using GraphPad Prism Version 8.0.0 for Windows.

### 4.4. Statistical Analysis

Data were reported as median and interquartile range (IQR). Paired comparisons have been performed by Wilcoxon signed-rank test. Statistical differences between two groups were assessed by Mann-Whitney U test. Statistical analysis was performed by SPSS (version 26, IBM SPSS Statistics). Graphics have been performed by KaleidaGraph software (version 4.5.4, Synergy, Reading, PA, USA). *p* values < 0.05 were considered as significant.

## Figures and Tables

**Table 1:** Summarizes the main clinical parameters of the three patients in our study.

Patient	Age	SCC (mEq/L)	Fecal Elastase (µg/g)	Last FEV_1_ (Liter, %)	Chronic Infection	CFTR-RD (Y/N)	OLTT (Y/N)
1 *	48	104	<100	0.65; 26	PA, BG	Y	Y
2 *	40	82	>500	1.25; 38	PA	N	Y
3	59	114	>500	0.59; 28	PA	N	Y

* These patients presented recurrent massive haemoptysis, not responding to bronchial artery embolization. Abbreviations: SCC: sweat chloride concentrations at CF diagnosis; FEV_1_: Forced Expiratory Volume in 1 s; CFTR-RD: CF related diabetes; PA: pseudomonas aeruginosa; BG: Burkholderia gladioli; OLTT: Oxygen Long-Term Therapy, N: Not, Y: Yes.

**Table 2:** Kaftrio^TM^ effect on CFTR-dependent Cl-secretion in CF and NO-CF nasal epithelial cells.

Type of Cells	No-Treated	Treated	*p* Value ^a^
CF HNEC (*n* = 6)	0.2 (0.2–0.3)	4.3 (3.8–5.0)	0.031
NO-CF HNEC (*n* = 12)	5.2 (4.0–5.9)	4.3 (3.7–5.7)	0.116
*p* value ^b^	<0.0001	0.964	

^a^ Wilcoxon signed-rank test; ^b^ Mann-Whitney U test. Significant values are reported in bold.

## Data Availability

Data sharing is not applicable to this article.
